# Comparison of clinical outcomes in hospitalized patients with COVID-19 or non-COVID-19 community-acquired pneumonia in a prospective observational cohort study

**DOI:** 10.1007/s15010-024-02292-z

**Published:** 2024-05-18

**Authors:** Hans-Jakob Meyer, Lukas Mödl, Olesya Unruh, Weiwei Xiang, Sarah Berger, Moritz Müller-Plathe, Gernot Rohde, Mathias W. Pletz, Jan Rupp, Norbert Suttorp, Martin Witzenrath, Thomas Zoller, Mirja Mittermaier, Fridolin Steinbeis, CAPNETZ study group, A Fuchs, M Engelmann, D Stolz, W Bauer, H. C Mücke, S Schmager, B Schaaf, J Kremling, D Nickoleit-Bitzenberger, H Azzaui, M Hower, F Hempel, K Prebeg, K Popkirova, M Kolditz, C Bellinghausen, A Grünewaldt, M Panning, T Welte, T Fühner, M. van’t Klooster, G Barten-Neiner, W Kröner, N Adaskina, F Eberherdt, C Julius, T Illig, N Klopp, B. T Schleenvoigt, C Forstner, A Moeser, J Ankert, D Drömann, P Parschke, K Franzen, N Käding, F Waldeck, C Spinner, J Erber, F Voit, J Schneider, D Heigener, I Hering, W Albrich, M Seneghini, F Rassouli, S Baldesberger, A Essig, S Stenger, M Wallner, H Burgmann, L Traby, L Schubert, R Chen

**Affiliations:** 1 Department of Infectious Diseases, Respiratory Medicine and Critical Care, Charité – Universitätsmedizin Berlin, Corporate Member of Freie Universität Berlin and Humboldt-Universität Zu Berlin, Charitéplatz 1, 10117 Berlin, Germany; 2 Department of Pneumology, Helios Klinikum Emil Von Behring, Lungenklinik Heckeshorn, Berlin, Germany; 3 Institute of Biometry and Clinical Epidemiology, Charité - Universitätsmedizin Berlin, Corporate Member of Freie Universität Berlin and Humboldt-Universität Zu Berlin, Berlin, Germany; 4 CAPNETZ STIFTUNG, Hannover, Germany; 5 Department of Respiratory Medicine, Goethe University, University Hospital, Medical Clinic I, Frankfurt/Main, Germany; 6 Biomedical Research in Endstage and Obstructive Lung Disease Hannover (BREATH), Member of the German Center for Lung Research (DZL), Hannover, Germany; 7 Institute of Infectious Diseases and Infection Control, Jena University Hospital /Friedrich Schiller University, Jena, Germany; 8 Department of Infectious Diseases and Microbiology, University Hospital Schleswig-Holstein, Lübeck, Germany; 9 German Center for Lung Research (DZL), Berlin, Germany; 10 Berlin Institute of Health at Charité – Universitätsmedizin Berlin, Berlin, Germany

**Keywords:** COVID-19, Community-acquired pneumonia, SARS-CoV-2, Observational cohort study

## Abstract

**Purpose:**

Coronavirus disease 2019 (COVID-19) and non-COVID-19 community-acquired pneumonia (NC-CAP) often result in hospitalization with considerable risks of mortality, ICU treatment, and long-term morbidity. A comparative analysis of clinical outcomes in COVID-19 CAP (C-CAP) and NC-CAP may improve clinical management.

**Methods:**

Using prospectively collected CAPNETZ study data (January 2017 to June 2021, 35 study centers), we conducted a comprehensive analysis of clinical outcomes including in-hospital death, ICU treatment, length of hospital stay (LOHS), 180-day survival, and post-discharge re-hospitalization rate. Logistic regression models were used to examine group differences between C-CAP and NC-CAP patients and associations with patient demography, recruitment period, comorbidity, and treatment.

**Results:**

Among 1368 patients (C-CAP: n = 344; NC-CAP: n = 1024), C-CAP showed elevated adjusted probabilities for in-hospital death (aOR 4.48 [95% CI 2.38–8.53]) and ICU treatment (aOR 8.08 [95% CI 5.31–12.52]) compared to NC-CAP. C-CAP patients were at increased risk of LOHS over seven days (aOR 1.88 [95% CI 1.47–2.42]). Although ICU patients had similar in-hospital mortality risk, C-CAP was associated with length of ICU stay over seven days (aOR 3.59 [95% CI 1.65–8.38]). Recruitment period influenced outcomes in C-CAP but not in NC-CAP. During follow-up, C-CAP was linked to a reduced risk of re-hospitalization and mortality post-discharge (aOR 0.43 [95% CI 0.27–0.70]).

**Conclusion:**

Distinct clinical trajectories of C-CAP and NC-CAP underscore the need for adapted management to avoid acute and long-term morbidity and mortality amid the evolving landscape of CAP pathogens.

**Supplementary Information:**

The online version contains supplementary material available at 10.1007/s15010-024-02292-z.

## Introduction

Community-acquired pneumonia (CAP) is among the most frequent causes of hospitalization worldwide and the associated mortality remains high [1, 2]. Coronavirus disease 2019 (COVID-19), caused by severe acute respiratory syndrome coronavirus 2 (SARS-CoV-2), has spread worldwide, increasing morbidity and mortality in most populations. The clinical spectrum of COVID-19 ranges from asymptomatic carriers to severe illness. Moderate and severe COVID-19 cases require hospitalization and are characterized by pneumonia as leading clinical feature [3]. The expansion of the SARS-CoV-2 pandemic to Central Europe in 2020 and the subsequent implementation of non-pharmacological interventions altered the spectrum of predominant pathogens causing CAP, placing SARS-CoV-2 among the primary pathogens of community-acquired pneumonia [4].

The outstanding characteristics of COVID-19 community-acquired pneumonia (C-CAP) compared to the non-COVID-19 CAP (NC-CAP) group are manifold: The pathophysiology of COVID-19, involving vascular activation [5], hypercoagulability [6], and the fibroproliferative activation of pulmonary macrophages [7], is one domain highlighting the disease’s peculiarity. Also on the clinical level, COVID-19 pneumonia is distinct from how CAP has been observed so far, e. g. considering the trajectory of severe disease: While severe bacterial CAP is often linked to a rapidly evolving respiratory failure and septic shock [8], respiratory failure in COVID-19 pneumonia develops more gradually [9]. In severe disease, both diagnoses are typically accompanied by complications resulting from intensive care unit (ICU) treatment, such as ventilator-associated pneumonia or catheter-associated bloodstream infections [10]. In the acute and convalescence phase of both COVID-19 and NC-CAP, an increase in cardiovascular events and cardiovascular mortality is observed [11, 12].

Contextualizing C-CAP outcomes with NC-CAP might help estimating the excess risk linked to C-CAP, e.g. in an emergency room or ICU setting. As for policymaking and resource allocation during pandemics, the expected length of hospital stay (LOHS) is a parameter of critical importance in estimating in-patient health-care demands [13]. This study presents data from two prospective observational cohorts of C-CAP and NC-CAP patients recruited following the same study protocol. We compare the risk for unfavorable hospitalization outcomes such as in-hospital mortality, ICU treatment, invasive mechanical ventilation (MV), vasopressor use, LOHS and length of ICU stay (ICULOS) between C-CAP and NC-CAP patients. Using data from a 180-days follow-up, we moreover compare risk for post-discharge mortality and morbidity (represented by the re-hospitalization rate) in the two groups. In congruence with experiences from now four years of COVID-19, we hypothesized that increased risk for in-hospital death and severe disease was associated with C-CAP. We furthermore suspected that C-CAP leads to prolonged LOHS and thus higher resource demand than NC-CAP. Considering follow-up outcomes, we conclusively assumed that post-discharge mortality and morbidity in C-CAP exceed those of NC-CAP patients.

## Methods

### Dataset

We analyzed data collected by the multi-national network CAPNETZ (Competence network community-acquired pneumonia) in the framework of the eponymous multinational prospective cohort study conducted in 35 Central European clinical centers (34 hospitals and one outpatient clinic), of which 30 were in Germany, two in Switzerland, and one each in Austria, Italy, and the Netherlands (https://capnetz.de/infrastruktur/). Recruitment and data collection followed the study protocols CAPNETZ 2.0 (January 2017 until June 2021) or CAPNETZ-PROVID (October 2020 until June 2021). CAPNETZ 2.0 is an updated version of the CAPNETZ study protocol [14]. CAPNETZ-PROVID is an amendment to CAPNETZ, affiliated with the consortium PROVID (Clinical, Molecular and Functional Biomarkers for Prognosis, Pathomechanisms and Treatment Strategies of COVID-19), which was established in 2020 as a response to the SARS-CoV-2 pandemic. Inclusion criteria for participation in CAPNETZ-PROVID were age of 18 years or older and a positive SARS-CoV-2 polymerase chain reaction (PCR) at screening visit. CAPNETZ-PROVID recruitment took place in 12 of the CAPNETZ study centers. CAPNETZ 2.0 and CAPNETZ-PROVID were approved by the Ethics Committee of Hannover Medical School (301–2008) and are registered at ClinicalTrials.gov (NCT02139163, NCT04952337). All participants or their legal guardian provided written informed consent for study participation.

### Study design

From both the CAPNETZ 2.0 and the CAPNETZ-PROVID datasets, patients analyzed in this study were required to have a diagnosis of COVID-19 CAP or NC-CAP, defined by the CAPNETZ 2.0 inclusion criteria (CAP criteria): (i) Presentation of at least one clinical sign or symptom of pneumonia (fever, cough, purulent sputum, or rales/crackles in pulmonary auscultation) at study enrollment, (ii) Pulmonary infiltrations found in chest imaging, and (iii) Exclusion of hospital-acquired pneumonia (assumed if the patients were not hospitalized during the last 28 days and if diagnosis of pneumonia was made within 48 h after hospitalization). Patients with severe immunosuppression (recent chemotherapy, neutropenia, recent systemic steroid therapy or history of solid organ or stem cell transplant) were excluded. To facilitate appropriate group assignment (C-CAP vs. NC-CAP), we excluded patients from the analysis if no SARS-CoV-2 PCR test was performed at study enrollment in patients after the pandemic onset or if SARS-CoV-2 ribonucleic acid detection first occurred during the hospitalization. According to the SARS-CoV-2 PCR result from screening visit, we assigned participants to the C-CAP or NC-CAP group. Baseline demographics, comorbidities, and administration of key medications (antibiotics, remdesivir, dexamethasone) during the hospital phase were documented. We only considered participants who were hospitalized at study inclusion and not transferred to another hospital. Patients who withdrew their written study participation during follow-up and whose datasets were incomplete regarding our study participation criteria or outcomes were excluded. The recruitment date was classified as pre-pandemic, first, second, and third wave according to the classification of pandemic phases proposed by Tolksdorf et al. [15]. Participants or their legal representatives were contacted 180 days after inclusion for follow-up. If no information on post-discharge vital status was obtained, participants were considered lost to follow-up.

### Outcomes and subgroups

Outcomes of the hospitalization phase were in-hospital death, ICU treatment, use of invasive mechanical ventilation (MV), vasopressor treatment, LOHS over seven days, and patient status on day 28 (discharged, hospitalized, or death during hospitalization). For patients treated on an ICU, we analyzed ICULOS, time from hospitalization to first ICU admission, length of invasive MV, and time to intubation. LOHS was analyzed in subgroups defined by level of care and survival status. Post-discharge follow-up outcomes were death and, in participants who completed the 180 days post-hospital admission follow-up, additional hospitalizations.

### Statistical analysis

Continuous and discrete variables are presented as median with interquartile range (IQR). Group differences were assessed using Mann–Whitney-U test for continuous/discrete variables and Fisher’s exact (in observed frequencies under five in one of the groups) or Chi^2^ test for categorical variables. Kruskal–Wallis test compared ordinal variables among groups. Adjusted odds ratios with a 95% confidence interval, calculated based on multivariate logistic regression, compared risks for the different outcomes associated with C-CAP and NC-CAP, adjusting for sex, age, BMI, and the five most frequent comorbidities from both groups. Multivariate logistic regression analyzed factors associated with in-hospital mortality, LOHS over seven days, and ICU treatment, adjusting for age, sex, BMI, recruitment period, the five most common comorbidities within each diagnosis group and, in C-CAP, the use of remdesivir, dexamethasone, and antibiotics. Bar plots and Kaplan–Meier curves serve to illustrate the development of patient status and length of hospital stay. Right censoring was undertaken for LOHS over 28 days. Log-rank test was used to examine significant group differences in time-to-event analysis. Statistical significance was assumed for p < 0.05. Missing values are reported in sTable 1. Analyses and visualizations were performed using RStudio (Version 4.1.2) with the R packages ‘survival’, ‘survminer’ and ‘ggplot2’ [16, 17].

## Results

### Study participants

**Fig. 1:**
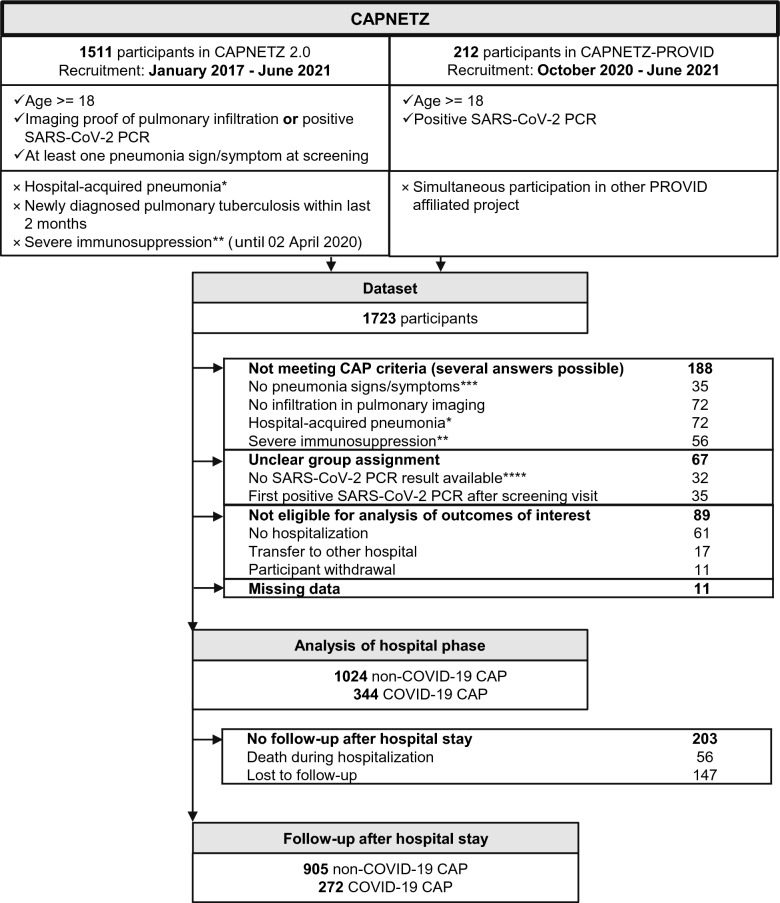
Patient flowchart. Ticks (✓) indicate inclusion criteria, crosses ( × ) exclusion criteria for CAPNETZ (left) and CAPNETZ-PROVID participation. *) Pneumonia onset ≥ 48 h after hospitalization or hospitalization during the last 28 days. **) Recent chemotherapy, neutropenia, recent systemic steroid therapy or history of solid organ or stem cell transplant ***) Fever, cough, purulent sputum, or rales/crackles in pulmonary auscultation at screening visit. ****) Applies after pandemic onset. CAPNETZ: competence network community-acquired pneumonia, PROVID: Clinical, Molecular and Functional Biomarkers for Prognosis, Pathomechanisms and Treatment Strategies of COVID-19, SARS-CoV-2: severe acute respiratory syndrome coronavirus 2, PCR: polymerase chain reaction, NC-CAP: non-COVID-19 community-acquired pneumonia, C-CAP: COVID-19 community-acquired pneumonia

Figure 1 depicts the participant flowchart in accordance with STROBE (Strengthening the Reporting of Observational studies in Epidemiology) recommendations [18]. The dataset included 1723 participants (1511 in CAPNETZ, 212 in CAPNETZ-PROVID). Exclusions (n = 355) were made based on CAP criteria, unclear group assignment, non-eligibility, or missing data. The remaining study sample (n = 1368) comprised 344 C-CAP and 1024 NC-CAP patients. The follow-up cohort (n = 1177) comprised 191 fewer patients, as 56 died during the initial hospital stay and 147 were lost to follow-up.

### Patient characteristics

Table 1 summarizes patient characteristics. C-CAP patients had lower age (60 vs. 66 years) and higher median body mass index (BMI, 27.4 kg/m^2^ vs. 25.7 kg/m^2^) than NC-CAP patients. Most C-CAP patients were recruited during the second wave of the pandemic (44.2%), while most NC-CAP patients were recruited before the pandemic (80.6%). The most common comorbidities in both groups were arterial hypertension (39.5% vs. 47.9%), diabetes mellitus (21.5% vs. 18.5%), atrial fibrillation (7.6% vs. 15.1%) and malignant diseases (8.1% vs. 17.1%), complemented by asthma in C-CAP (7.3%) and chronic obstructive pulmonary disease in NC-CAP (22.6%). Antibiotics were used in about half of the C-CAP patients and in most NC-CAP patients (51.2% vs. 98.5%). Among C-CAP patients, 19.5% received antiviral treatment with remdesivir and 45.6% anti-inflammatory treatment with dexamethasone. sTable 2 summarizes patient characteristics during the pandemic waves according to Tolksdorf et al. [15] Regarding C-CAP treatment throughout the pandemic waves, the use of antibiotics decreased (60.5% vs. 46.7% vs. 44.1%, p = 0.0323) while use of dexamethasone increased (17.7% vs. 55.3% vs. 75.0%, p < 0.0001).

**Table 1:** Demographics, comorbidities, and clinical characteristics of patients with COVID -19 disease community-acquired pneumonia (C-CAP) and non-COVID-19 community-acquired pneumonia (NC-CAP)

	NC-CAP	C-CAP	p-value
Total (n)	1024	344	
Demography			
Age (y) median (IQR)	66 (53–77)	60 (49–70)	** < 0.0001**
Sex female (%)	356 (34.8)	127 (36.9)	0.5107
BMI (kg/m^2^) median (IQR)*	25.7 (22.8–29.6)	27.4 (24.9–31.8)	** < 0.0001**
Recruitment phase			
Pre-pandemic	825 (80.6)	0 (0.0)	** < 0.0001**
First wave	122 (11.9)	124 (36.0)	
Second wave	37 (3.6)	152 (44.2)	
Third wave	40 (3.9)	68 (19.8)	
Comorbidities and lifestyle factors			
Arterial hypertension (%)	490 (47.9)	136 (39.5)	**0.0089**
Atrial fibrillation (%)	155 (15.1)	26 (7.6)	**0.0005**
Pre-existing heart failure (%)	63 (6.2)	8 (2.3)	**0.0086**
Coronary heart disease (%)	120 (11.7)	18 (5.2)	**0.0008**
COPD (%)	231 (22.6)	13 (3.8)	** < 0.0001**
Asthma (%)	71 (6.9)	25 (7.3)	0.9301
Diabetes mellitus (%)	189 (18.5)	74 (21.5)	0.2441
Hypercholesterinemia (%)*	135 (13.2)	21 (6.2)	**0.0007**
Malignant disease (%)	175 (17.1)	28 (8.1)	**0.0001**
Liver disease (%)	32 (3.1)	4 (1.2)	0.0516
Chronic kidney disease (%)	132 (12.9)	19 (5.5)	**0.0002**
Neurological disease (%)	105 (10.3)	21 (6.1)	**0.0282**
Autoimmune disease (%)	65 (6.3)	11 (3.2)	**0.0384**
HIV-positive (%)	45 (4.4)	4 (1.2)	**0.0038**
Smoking history (%)*	666 (65.7)	97 (30.5)	** < 0.0001**
Laboratory parameters at hospital admission			
WBC (count/nl) median (IQR)*	11.3 (8.3–15.4)	6.0 (4.7–8.8)	** < 0.0001**
CRP (mg/l) median (IQR)*	126.2 (55.0–218.2)	62.1 (26.7–109.5)	** < 0.0001**
PCT ≥ 0.5 ng/ml (%)*	168 (41.0)	26 (9.3)	** < 0.0001**
Lactate ≥ 20 mg/dl (%)*	34 (17.2)	25 (13.6)	0.4082
LDH ≥ 250 U/l (%)*	309 (47.6)	234 (81.8)	** < 0.0001**
Treatment during hospitalization			
Antibiotic (%)	1009 (98.5)	176 (51.2)	** < 0.0001**
Remdesivir (%)	0 (0.0)	67 (19.5)	** < 0.0001**
Dexamethasone (%)	6 (0.6)	157 (45.6)	** < 0.0001**

Bold numbers indicate p-values < 0.05

Asterisks (*) mark items with missing values as reported in sTable 1.

*IQR* inter-quartile range, *COPD* chronic obstructive pulmonary disease, *HIV* human immunodeficiency virus, *WBC* white blood cells, *CRP* C-reactive protein, *PCT* procalcitonin, *LDH* lactate dehydrogenase

### Hospitalization outcomes

**Fig. 2:**
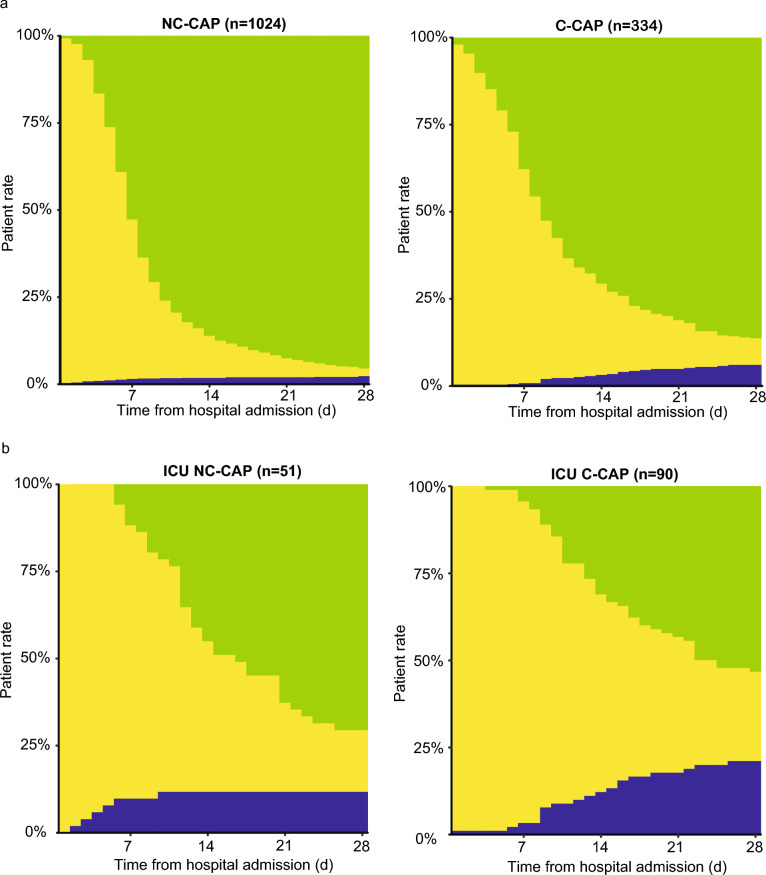
Distribution of hospitalization status from admission until 28 days after hospitalization in all patients and ICU patients with C-CAP or NC-CAP. X-axes depict time (d) from hospitalization, Y-axes the rate of patients (%). Green bars represent the percentage of discharged patients, yellow bars the percentage of hospitalized patients, and blue bars the percentage of participants who died in the hospital. The right plots describe the trajectory of the C-CAP participants; the left plots of the NC-CAP participants. ICU: intensive care unit, NC-CAP: non-COVID-19 community-acquired pneumonia, C-CAP: COVID-19 community-acquired pneumonia

Table 2 details the hospitalization outcomes. The C-CAP group was at higher risk for in-hospital death than NC-CAP (7.6% vs. 2.9%, p = 0.0003, aOR 4.48 [95% CI 2.38–8.53]). ICU treatment risk was elevated in C-CAP compared to NC-CAP (26.2% vs. 5.0%, p < 0.0001, aOR 8.08 [95% CI 5.31–12.52]). C-CAP was linked to a higher rate of LOHS over seven days than NC-CAP (61.3% vs. 45.7%, p < 0.0001, aOR 2.21 [95% CI 1.67–2.92]). LOHS over 28 days was more prevalent in C-CAP (7.6% vs. 2.1%, p < 0.0001, aOR 4.56 [95% CI 2.35–8.97]). The 28-day trajectory of patient statuses (hospitalized vs. discharged alive vs. in-hospital death) is depicted in Fig. 2a. In C-CAP patients, in-hospital death occurred less frequently during the first wave than in the second and third wave (3.2% vs. 11.2% vs. 7.4%, p = 0.0451, sTable 3). Throughout all waves, rate of ICU treatment in C-CAP patients was comparable (22.6% vs. 25.7% vs. 33.8%, p = 0.2335). Median LOHS of C-CAP patients was highest during the first pandemic wave (11 d [7, 8, 9, 10, 11, 12, 13, 14, 15, 16, 17] vs. 8 d [5, 6, 7, 8, 9, 10, 11, 12] vs. 9 d [6, 7, 8, 9, 10, 11, 12, 13], p = 0.0031), leading to a higher share of patients hospitalized longer than seven days (71.8% vs. 56.6% vs. 52.9%, p = 0.0102). No significant differences were observed in NC-CAP patients between periods before and during the pandemic (sTable 4).

**Table 2:** Hospitalization outcomes of patients with COVID-19 community-acquired pneumonia (C-CAP) and non-COVID-19 community-acquired pneumonia (NC-CAP)

	NC-CAP	C-CAP	p-value	aOR (95% CI)
Total (n)	1024	344		
In-hospital death (%)	30 (2.9)	26 (7.6)	**0.0003**	**4.48 (2.38–8.53)**
ICU treatment (%)	51 (5.0)	90 (26.2)	** < 0.0001**	**8.08 (5.31–12.52)**
Invasive MV (%)	12 (1.2)	28 (8.1)	** < 0.0001**	**9.11 (4.26–20.89)**
Vasopressor treatment (%)	14 (1.4)	33 (9.6)	** < 0.0001**	**10.49 (5.17–22.65)**
LOHS > 7 d (%)	468 (45.7)	211 (61.3)	** < 0.0001**	**2.21 (1.67–2.92)**
LOHS > 28 d (%)	22 (2.1)	26 (7.6)	** < 0.0001**	**4.56 (2.35–8.97)**
LOHS (d) median (IQR)	7 (5–10)	9 (6–15)	** < 0.0001**	

Bold numbers indicate p-values < 0.05

Adjusted odds ratios were calculated using age, sex, and the most frequent five comorbidities of both groups as covariates

*IQR* inter-quartile range, *aOR* adjusted odds ratio, *CI* confidence interval, *MV* mechanical ventilation, *LOHS* length of hospital stay, *ICU* intensive care unit

### ICU patient outcomes

Hospitalization outcomes of ICU-treated patients are summarized in Table 3. Risk of in-hospital death was comparable between C-CAP and NC-CAP ICU patients (26.7% vs. 19.6%, p = 0.4613, aOR 3.08 [95% CI 0.83–12.86]). Risks of invasive MV (31.9% vs. 23.5%, p = 0.4442, aOR 1.42 [95% CI 0.53–4.11]) and vasopressor use (36.7% vs. 27.5%, p = 0.3526, aOR 1.90 [0.70–5.50]) were similar. C-CAP patients with ICU treatment were more likely to exceed seven days of LOHS (92.2% vs. 78.4%, aOR 6.01 [95% CI 1.47–28.83]) and had significantly higher risk for ICULOS over seven days (46.7% vs. 19.6%, aOR 3.36 [95% CI 1.28–9.64]). Median time until ICU admission (one day) and intubation (four days) were equal in C-CAP and NC-CAP. The 28-day trajectory of patient statuses is depicted in Fig. 2b.

**Table 3:** Hospitalization outcomes of intensive care unit treated patients with COVID-19 community-acquired pneumonia (ICU C-CAP) and non-COVID-19 community-acquired pneumonia (ICU NC-CAP)

	NC-CAP	C-CAP	p-value	aOR (95% CI)
Total (n)	51	90	.	.
In-hospital death (%)	10 (19.6)	24 (26.7)	0.4613	3.08 (0.83–12.86)
Invasive MV (%)	12 (23.5)	28 (31.1)	0.4442	1.42 (0.53–4.11)
Vasopressor treatment (%)	14 (27.5)	33 (36.7)	0.3526	1.90 (0.70–5.50)
LOHS > 7 d (%)	40 (78.4)	83 (92.2)	**0.0361**	**6.01 (1.47–28.83)**
ICULOS > 7 d (%)	10 (19.6)	42 (46.7)	**0.0025**	**3.36 (1.28–9.64)**
LOHS (d) median (IQR)	13 (9–22)	17 (11–29)	**0.0475**	.
ICULOS (d) median (IQR)	3 (1–7)	7 (4–15)	** < 0.0001**	.
Length of invasive mechanical ventilation (d) median (IQR)	4 (1–8)	15 (8–23)	**0.0115**	.
Length of vasopressor treatment (d) median (IQR)	2 (1–8)	10 (2–22)	**0.0127**	.
Time from hospital admission to ICU admission (d) median (IQR)	1 (0–3)	1 (0–3)	0.9266	.
Time from hospital admission to first intubation (d) median (IQR)	4 (2–10)	4 (3–7)	0.9055	.
Time from ICU admission to first intubation (d) median (IQR)	1 (0–1)	1 (0–1)	0.4755	.

Bold numbers indicate p-values < 0.05

*IQR* inter-quartile range, *CI* confidence interval, *ICU* intensive care unit, *MV* mechanical ventilation, *LOHS* length of hospital stay, *ICULOS* length of ICU stay

### Risk factor analysis

sTable 5 presents adjusted odds ratios from multivariate logistic regression with patient demography, recruitment period, comorbidity, and treatment as covariates for in-hospital death, LOHS over seven days, and ICU treatment. Female sex was associated with lower risk for ICU treatment in C-CAP (aOR 0.36 [95% CI 0.18–0.68]) but showed no association in NC-CAP (aOR 0.76 [95% CI 0.38–1.43]). Higher age was linked to elevated risks for in-hospital death and LOHS over seven days in both groups. In C-CAP, the second pandemic wave was linked to higher risk of in-hospital mortality (aOR 7.64 [95% CI 1.58–60.60]). Both the second and third pandemic wave were associated with lower risk of LOHS over seven days in C-CAP (second: aOR 0.28 [95% CI 0.13–0.58]; third: aOR 0.24 [95% CI 0.11–0.55]). In NC-CAP, recruitment period was not associated with the outcomes. Administration of antibiotics as well as dexamethasone were associated with LOHS over seven days and ICU treatment in C-CAP.

### Subgroup analysis of LOHS

**Fig. 3:**
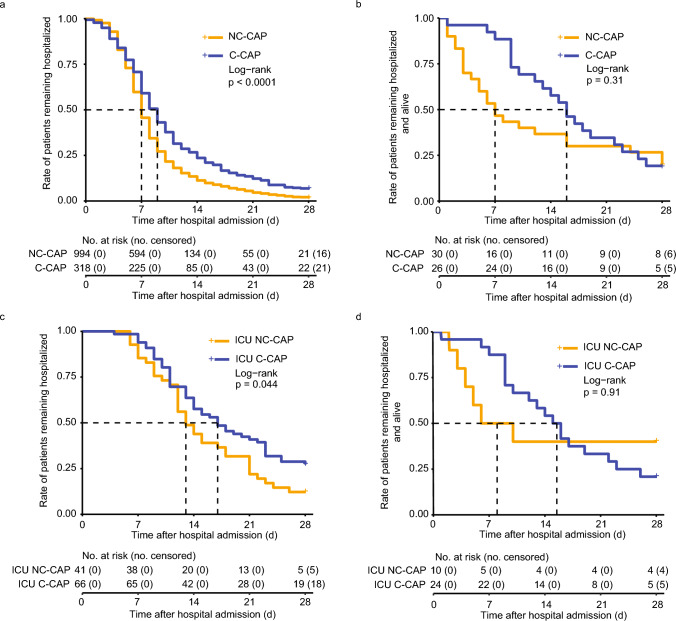
Time-to-event analysis of length of hospital stay. X-axes represent time (days) after hospital admission, Y-axes the rate of patients remaining hospitalized. Blue curves represent participants with COVID-19 CAP, orange curves non-COVID-19 CAP. Plus ( + ) sign indicates censoring (at day 28 after hospital admission). Dashed lines represent median length of hospital stay. a) Time from hospital admission to hospital discharge in survivors of hospitalization. b) Time from hospital admission to in-hospital death in participants who deceased during the hospital stay. c) Time from hospital admission to hospital discharge in ICU (intensive care unit) treated survivors of hospitalization. d) Time from admission to in-hospital death in ICU-treated non-survivors of hospitalization. NC-CAP: non-COVID-19 community-acquired pneumonia, C-CAP: COVID-19 community-acquired pneumonia. No: number

Figure 3 presents Kaplan–Meier curves of LOHS in C-CAP and NC-CAP. C-CAP patients who survived the hospitalization had a longer median LOHS than NC-CAP patients who were discharged alive (9 d vs. 7 d, p < 0.0001, Fig. 3a). In patients who deceased during hospitalization, median LOHS in C-CAP was longer than NC-CAP (16 d vs. 7 d), however remained below the assumed level of significance (p = 0.3054, Fig. 3b). In surviving ICU patients, median LOHS was longer in C-CAP than in NC-CAP (17 d vs. 13 d, p = 0.0441, Fig. 3c). In deceased ICU patients, median LOHS was longer in C-CAP than in NC-CAP (16 d vs. 8 d), however the difference remained under the assumed level of significance (p = 0.9056, Fig. 3d).

### Follow-up

Outcomes from the 180-day follow-up are presented in sTable 6. 272 (79.1%) C-CAP patients and 905 (88.3%) NC-CAP patients were included in the 180 days post-hospitalization follow-up. Fewer C-CAP patients died after hospital discharge than NC-CAP patients (1.5% vs. 3.9%, p = 0.0540, aOR 0.65 [95% CI 0.11–1.99]). C-CAP patients were at a lower risk for re-hospitalization than NC-CAP patients (9.3% vs. 20.1%, p < 0.0001, aOR 0.43 [95% CI 0.27–0.70]).

## Discussion

This study analyzed prospective cohort study data on hospitalized patients with C-CAP and NC-CAP, providing insights into clinical outcomes both during hospitalization and a subsequent follow-up. Spanning five countries and 35 clinical centers over 54 months, the findings underscore the enormous severity in COVID-19 pneumonia during the early stages of the pandemic. The results portray the temporal spectrum of COVID-19 pneumonia.

treatment, trajectory, and outcomes during the peak of a global health emergency, and put them into context with NC-CAP.

The analysis revealed a four-fold higher risk for in-hospital death, an eight-fold higher risk of ICU treatment, and two-fold higher risk of hospitalization exceeding seven days in C-CAP compared to NC-CAP patients. Underlining this, C-CAP patients’ median length of invasive MV exceeded NC-CAP by eleven days. In cases of in-hospital death, C-CAP patients’ treatment duration was more than double than in NC-CAP. This highlights an exceptional burden of severe illness and elevated healthcare demand in C-CAP compared to NC-CAP, particularly among ICU-treated patients, whose risk of remaining hospitalized over seven days was six-fold higher in C-CAP. We observed variability in C-CAP outcomes across pandemic phases, with longer hospital stays in the first wave, but higher risk of in-hospital death during the second and third wave. Having reported excess LOHS during the first wave compared to second and third wave resembling our results, investigators from Bologna assigned this to changing containment policies and improved clinical management during the later stages of the pandemic [19]. Additionally, Lampl et al. discussed caution regarding hospital admission of COVID-19 cases among both patients and clinics during the later stages of the pandemic, potentially leading to delayed hospitalizations and, consequently, to shorter hospital stays either through discharge or due to in-hospital mortality as in our study [20].

COVID-19 case fatality rates and mortality throughout the pandemic were shaped by the emergence of evolving virus variants, improved treatment guidelines, the demographic composition, including age structure and comorbidities among COVID-19 cases, as well as the rising rates of immunization [21, 22]. Supporting our results, Lampl et al. reported the highest COVID-19 case fatality rate in the Regensburg area during the second COVID-19 wave, attributing this to the spreading of the disease to an older-aged population during late 2020 and 2021, where a strained health-care system disposed over limited resources and effective SARS-CoV-2 vaccines were not yet broadly available [20]. Interestingly, there were no significant differences in NC-CAP outcomes comparing the recruitment phases before and during the pandemic in our study, emphasizing the distinct impact of the pandemic evolution on C-CAP outcomes.

Antibiotic treatment in C-CAP was associated with ICU treatment, in-hospital death, and excess LOHS in our cohort. Of note, though in our cohort the use of antibiotics in C-CAP decreased from the first to the third wave patients, mortality increased. We assume that this seemingly paradoxical relationship is caused by improved guidelines for antibiotic use in COVID-19 based on the observation that only less than 10% and especially ICU patients had bacterial superinfection during the early stages of the pandemic [23].

In the multivariate analyses of factors associated with in-hospital mortality, LOHS over seven days, and ICU treatment, we observed further differences between C-CAP and NC-CAP: Notably, female sex in C-CAP was independently associated with lower risk of ICU treatment, while sex had no significant impact on the outcomes in NC-CAP. The roles of sex and gender in COVID-19 have been extensively discussed: While gender-associated disparities in lifestyle, profession, and the resulting risk of SARS-CoV-2 transmission were crucial for the higher incidence and disease severity in male gender during the early stages of the pandemic [24], sex-determined differences are a major factor influencing a patient’s immune response to SARS-CoV-2 with different mechanisms of acute deterioration [25]. In contrast to our findings, a systematic review highlighted worse outcomes for men also in NC-CAP [26]. More research is needed to distinguish biological and social determinants for unfavorable outcomes in CAP.

These findings are an important contribution to existing results from retrospective and registry analyses attempting a contextualization of C-CAP among CAP. In line with our results, Cangemi et al. found in a prospective cohort study that COVID-19 was associated with a five-fold increase in the in-hospital mortality rate compared to NC-CAP [6]. In a retrospective analysis of hospitalized patients with COVID-19 or Influenza A from a nation-wide hospital network in Germany, Kodde et al. found that COVID-19 was associated with three-fold increased odds for in-hospital death than Influenza A patients [27]. Serrano Fernandez et al. observed rates of in-hospital death and invasive MV twice as high in COVID-19 pneumonia than in bacteremic pneumococcal CAP [28].

In our study, C-CAP was associated with a lower risk of recurrent hospitalization in a 180-days follow up than NC-CAP. This finding is in line with Novelli et al. [29], suggesting that long-term morbidity in COVID-19 depends less on the initial disease severity, but more on patients’ baseline morbidity. Nevertheless, it is important to note that post-discharge morbidity in COVID-19 can manifest in the diverse features of the post-COVID-19 syndrome, characterized by long-lasting fatigue, respiratory and neurocognitive symptoms, and pulmonary function impairment [30], and does not necessarily lead to re-hospitalization. As we did not evaluate the occurrence of long-lasting symptoms, we cannot draw conclusions regarding the post-COVID-19 syndrome and comparable features in NC-CAP. To improve our understanding of morbidity after hospitalization for community-acquired pneumonia, prospective studies like the German national pandemic cohort network NAPKON, evaluating symptoms, pathophysiology, and ideally interventional measures, are desperately needed [31].

Strengths of our analysis are the prospective, multi-national dataset and the harmonized definition of the study participation criteria, yielding a highly comparable sample of both C-CAP and NC-CAP patients examined under the same study protocol for in-hospital and post-discharge outcomes. This analysis provides a comprehensive report of hospitalization and follow-up outcomes comparing both disease groups, offering a retrospective contextualization of the pandemic’s impact on the spectrum of CAP patients. The data show the diverging disease trajectories in C-CAP and NC-CAP and how both treatment and outcomes in C-CAP changed chronologically with the progression of the pandemic.

This study has limitations. The total number of eligible patients is unknown, introducing potential selection bias. E. g., patients with severe C-CAP immediately intubated upon hospital arrival may not have been included, possibly impacting the results. However, time to intubation did not differ between C-CAP and NC-CAP ICU patients, supporting the data validity. Seventeen study participants were excluded, as they were transferred to another hospital. This could introduce a referral bias, particularly as cases with complex disease trajectories, such as those needing extracorporeal membrane oxygenation therapy or extended weaning from mechanical ventilation, are more likely to be referred to specialized hospitals. Furthermore, our analysis focused on in-hospital death, ICU treatment, and LOHS as indicators for disease severity. A more detailed classification of pneumonia outcomes, e. g. varying levels of respiratory support such as high flow nasal oxygen or extra-corporal membrane oxygenation, was not feasible in our data sample. Future large-scale prospective studies need to address CAP severity degrees in more detail.

## Conclusion

Based on data from a multinational prospective cohort, this analysis shows the excess risk of C-CAP patients from the first three pandemic waves for in-hospital death, ICU treatment, and prolonged hospital and ICU stay compared to NC-CAP. Risk of re-hospitalization after discharge was elevated in NC-CAP, highlighting the role of CAP etiology in acute and chronic morbidity.

## Supplementary Information

Below is the link to the electronic supplementary material.

Supplementary file1 (DOCX 45 KB)
