# Supplementary material for: Comparison of clinical outcomes in hospitalized patients with COVID-19 or non-COVID-19 community-acquired pneumonia in a prospective observational cohort study
Source: Infection. 2024 May 18;52(6):2359–70. doi: 10.1007/s15010-024-02292-z (PMC11621138; doi:10.1007/s15010-024-02292-z)
Supplement: Supplementary file 1 — Supplementary file1 (DOCX 45 KB) [file 15010_2024_2292_MOESM1_ESM.docx]

**Comparison of clinical outcomes in hospitalized patients with COVID-19 or non-COVID-19 community-acquired pneumonia in a prospective observational cohort study**

**Authors and affiliations:**

Hans-Jakob Meyer^1,2^, Lukas Mödl^3^, Olesya Unruh^4^, Weiwei Xiang^1^, Sarah Berger^1^, Moritz Müller-Plathe^1^, Gernot Rohde^4,5,6^, Mathias W. Pletz^4,7^, Jan Rupp^4,8^, Norbert Suttorp^1,4,9^, Martin Witzenrath^1,4,9^, Thomas Zoller^1^, Mirja Mittermaier^1,10*^, Fridolin Steinbeis^1*^, CAPNETZ study group

**Corresponding Author:**

Fridolin Steinbeis MD

Department of Infectious Diseases, Respiratory Medicine and Critical Care

Charité – Universitätsmedizin Berlin

Charitéplatz 1, 10117 Berlin

E-mail: fridolin.steinbeis@charite.de

Phone: +49 30 450 665134

^1^ Charité – Universitätsmedizin Berlin, Corporate Member of Freie Universität Berlin and Humboldt-Universität zu Berlin, Department of Infectious Diseases, Respiratory Medicine and Critical Care, Berlin, Germany

^2^ Department of Pneumology, Lungenklinik Heckeshorn, Helios Klinikum Emil von Behring, Berlin, Germany

^3^ Charité - Universitätsmedizin Berlin, Corporate Member of Freie Universität Berlin and Humboldt-Universität zu Berlin, Institute of Biometry and Clinical Epidemiology, Berlin, Germany

^4^ CAPNETZ STIFTUNG, Hannover, Germany

^5^ Goethe University Frankfurt, University Hospital, Medical Clinic I, Department of Respiratory Medicine, Frankfurt/Main, Germany

^6^ Biomedical Research in Endstage and Obstructive Lung Disease Hannover (BREATH), Member of the German Center for Lung Research (DZL)

^7^ Institute of Infectious Diseases and Infection Control, Jena University Hospital /Friedrich Schiller University, Jena, Germany

^8^ Department of Infectious Diseases and Microbiology, University Hospital Schleswig-Holstein, Lübeck, Germany

^9^ German Center for Lung Research (DZL), Berlin, Germany

^10^ Berlin Institute of Health at Charité – Universitätsmedizin Berlin, Berlin, Germany.

**Supplementary material**

**sTable 1** Missing values on baseline characteristics and admission findings

|  | **NC-CAP** | **C-CAP** |
| --- | --- | --- |
| Age | 0.00% | 0.00% |
| Sex female | 0.00% | 0.00% |
| Body mass index | 3.22% | 9.88% |
| Recruitment phase | 0.00% | 0.00% |
| Arterial hypertension | 0.00% | 0.00% |
| Atrial fibrillation | 0.00% | 0.00% |
| Pre-existing heart failure | 0.00% | 0.00% |
| Coronary heart disease | 0.00% | 0.00% |
| COPD | 0.00% | 0.00% |
| Asthma | 0.00% | 0.00% |
| Diabetes mellitus | 0.00% | 0.00% |
| Hypercholesterinemia | 0.39% | 2.33% |
| Malignant disease | 0.00% | 0.00% |
| Liver disease | 0.00% | 0.00% |
| Chronic kidney disease | 0.00% | 0.00% |
| Neurological disease | 0.00% | 0.00% |
| Autoimmune disease | 0.00% | 0.00% |
| HIV-positive | 0.00% | 0.00% |
| Smoking history | 0.00% | 0.00% |
| WBC | 2.05% | 1.45% |
| CRP | 3.52% | 2.03% |
| PCT | 59.96% | 18.31% |
| Lactate | 80.66% | 46.51% |
| LDH | 36.62% | 16.86% |
| Antibiotic | 0.00% | 0.00% |
| Remdesivir | 0.00% | 0.00% |
| Dexamethasone | 0.00% | 0.00% |

Bold values indicate baseline characteristics and admission findings with missing values. C-CAP. COVID-19 community-acquired pneumonia, NC-CAP: non-COVID-19 community-acquired pneumonia, COPD: chronic obstructive pulmonary disease, HIV: human immunosufficiency virus, WBC: white blood cells, CRP: C-reactive protein, PCT: procalcitonin, LDH: lactate dehydrogenase.

**sTable 2** Demographics, comorbidities, and clinical characteristics of patients with COVID-19 community-acquired pneumonia (C-CAP) and non-COVID-19 community-acquired pneumonia (NC-CAP) divided by recruitment phase according to Tolksdorf et al.

|  | **NC-CAP** | |  | **C-CAP** | | |  |
| --- | --- | --- | --- | --- | --- | --- | --- |
|  | **Before pandemic** | **During pandemic** | **p-value** | **First wave** | **Second wave** | **Third wave** | **p-value** |
| Total (n) | 825 | 199 | .. | 124 | 152 | 68 | .. |
| **Demography** | | | | | | | |
| Age (y) median (IQR) | 66.0 (53.0 - 77.0) | 67.0 (56.0 - 79.0) | **0.0265** | 58.5 (46.0 - 70.0) | 61.0 (53.0 - 72.0) | 59.0 (48.0 - 68.2) | 0.1352 |
| Sex female (%) | 290 (35.2) | 66 (33.2) | 0.6563 | 46 (37.1) | 60 (39.5) | 21 (30.9) | 0.4743 |
| BMI (kg/m²) median (IQR)* | 25.7 (22.7 - 29.4) | 25.9 (23.1 - 30.2) | 0.3898 | 26.1 (24.2 - 30.2) | 27.6 (25.1 - 31.9) | 28.4 (26.0 - 32.9) | **0.0317** |
| **Comorbidities and lifestyle factors** | | | | | | | |
| Arterial hypertension (%) | 397 (48.1) | 93 (46.7) | 0.7851 | 45 (36.3) | 65 (42.8) | 26 (38.2) | 0.5334 |
| Atrial fibrillation (%) | 120 (14.5) | 35 (17.6) | 0.3347 | 11 (8.9) | 11 (7.2) | 4 (5.9) | 0.7402 |
| Pre-existing heart failure (%) | 50 (6.1) | 13 (6.5) | 0.9327 | 1 (0.8) | 5 (3.3) | 2 (2.9) | 0.3789 |
| Coronary heart disease (%) | 99 (12.0) | 21 (10.6) | 0.6549 | 6 (4.8) | 8 (5.3) | 4 (5.9) | 0.9527 |
| COPD (%) | 198 (24.0) | 33 (16.6) | **0.0314** | 5 (4.0) | 4 (2.6) | 4 (5.9) | 0.4679 |
| Asthma (%) | 58 (7.0) | 13 (6.5) | 0.9262 | 15 (12.1) | 6 (3.9) | 4 (5.9) | **0.0336** |
| Diabetes mellitus type 1/2(%) | 153 (18.5) | 36 (18.1) | 0.9627 | 22 (17.7) | 34 (22.4) | 18 (26.5) | 0.3499 |
| Hypercholesterinemia (%)* | 103 (12.5) | 32 (16.2) | 0.2162 | 4 (3.3) | 6 (4.1) | 11 (16.2) | **0.0026** |
| Malignant disease (%) | 131 (15.9) | 44 (22.1) | **0.0464** | 8 (6.5) | 17 (11.2) | 3 (4.4) | 0.1636 |
| Liver disease (%) | 24 (2.9) | 8 (4.0) | 0.5609 | 1 (0.8) | 3 (2.0) | 0 (0.0) | 0.5411 |
| Chronic kidney disease (%) | 107 (13.0) | 25 (12.6) | 0.9714 | 5 (4.0) | 10 (6.6) | 4 (5.9) | 0.6785 |
| Neurological disease (%) | 75 (9.1) | 30 (15.1) | **0.0179** | 5 (4.0) | 14 (9.2) | 2 (2.9) | 0.1173 |
| Autoimmune disease (%) | 52 (6.3) | 13 (6.5) | 1.0000 | 3 (2.4) | 7 (4.6) | 1 (1.5) | 0.5599 |
| HIV-positive (%) | 38 (4.6) | 7 (3.5) | 0.6314 | 3 (2.4) | 1 (0.7) | 0 (0.0) | 0.4170 |
| Smoking history (%)* | 541 (65.9) | 125 (64.8) | 0.8314 | 30 (26.8) | 44 (31.9) | 23 (33.8) | 0.5466 |
| **Laboratory parameters at hospital admission** | | | | | | | |
| WBC (count/nl) median (IQR)* | 11.4 (8.5 - 15.8) | 10.5 (8.0 - 14.6) | 0.0319 | 5.7 (4.8 - 7.6) | 6.0 (4.4 - 9.5) | 7.0 (5.1 - 9.3) | 0.1942 |
| CRP (mg/l) median (IQR)* | 134.0 (63.2 - 233.4) | 84.9 (31.6 - 165.0) | **<0.0001** | 58.0 (21.9 - 113.5) | 68.4 (28.4 - 111.3) | 54.4 (28.0 - 87.3) | 0.4657 |
| PCT ≥ 0.5 ng/ml (%)* | 122 (43.4) | 46 (35.7) | 0.1691 | 11 (10.9) | 12 (9.7) | 3 (5.4) | 0.5062 |
| Lactate ≥ 20 mg/dl (%)* | 17 (13.3) | 17 (24.3) | 0.0774 | 2 (3.4) | 13 (14.1) | 10 (29.4) | **0.0021** |
| LDH ≥ 250 U/l (%)* | 234 (46.1) | 75 (53.2) | 0.1603 | 79 (76.0) | 107 (82.9) | 48 (90.6) | 0.0730 |
| **Treatment during hospitalization** | | | | | | | |
| Antibiotic (%) | 817 (99.0) | 192 (96.5) | **0.0184** | 75 (60.5) | 71 (46.7) | 30 (44.1) | **0.0323** |
| Remdesivir (%) | 0 (0.0) | 0 (0.0) | 1.0000 | 28 (22.6) | 32 (21.1) | 7 (10.3) | 0.0974 |
| Dexamethasone (%) | 2 (0.2) | 4 (2.0) | 1.0000 | 22 (17.7) | 84 (55.3) | 51 (75.0) | **<0.0001** |

Asterisks (*) mark items with missing values as reported in sTable 1. IQR: inter-quartile range, BMI: body mass index, COPD: chronic obstructive pulmonary disease, HIV: human immunodeficiency virus, WBC: white blood cells, PCT: procalcitonin, LDH: lactate dehydrogenase.

|  | **First wave** | **Second wave** | **Third wave** | **p-value** |
| --- | --- | --- | --- | --- |
| Total (n) | 124 | 152 | 68 | .. |
| In-hospital death (%) | 4 (3.2) | 17 (11.2) | 5 (7.4) | **0.0451** |
| ICU treatment (%) | 28 (22.6) | 39 (25.7) | 23 (33.8) | 0.2335 |
| Invasive MV (%) | 11 (8.9) | 13 (8.6) | 4 (5.9) | 0.7457 |
| Vasopressor treatment (%) | 12 (9.7) | 16 (10.5) | 5 (7.4) | 0.7607 |
| LOHS (d) median (IQR) | 11(7 - 17) | 8 (5 - 12) | 9 (6 - 13) | **0.0031** |
| LOHS > 7 d | 89 (71.8) | 86 (56.6) | 36 (52.9) | **0.0102** |
| LOHS > 28 d | 14 (11.3) | 8 (5.3) | 4 (5.9) | 0.1429 |

**sTable 3** Hospitalization outcomes of patients with COVID-19 community-acquired pneumonia (C-CAP) according to the recruitment phase.

Bold numbers indicate p-values <0.05. IQR: inter-quartile range, MV: mechanical ventilation, LOHS: length of hospital stay, ICU: intensive care unit.

**sTable 4** Hospitalization outcomes of patients with non-COVID-19 community-acquired pneumonia (NC-CAP) according to the recruitment phase according to Tolksdorf et al.

|  | **Before pandemic** | **During pandemic** | **p-value** |
| --- | --- | --- | --- |
| Total (n) | 825 | 199 | .. |
| In-hospital death (%) | 20 (2.4) | 10 (5.0) | 0.0857 |
| ICU treatment (%) | 36 (4.4) | 15 (7.5) | 0.0957 |
| Invasive MV (%) | 8 (1.0) | 4 (2.0) | 0.3914 |
| Vasopressor treatment (%) | 9 (1.1) | 5 (2.5) | 0.2262 |
| LOHS (d) median (IQR) | 7 (5 - 10) | 7 (5 - 10) | 0.1700 |
| LOHS > 7 d | 385 (46.7) | 83 (41.7) | 0.2376 |
| LOHS > 28 d | 13 (1.6) | 9 (4.5) | **0.0214** |

Bold numbers indicate p-values <0.05. IQR: inter-quartile range, MV: mechanical ventilation, LOHS: length of hospital stay, ICU: intensive care unit.

**sTable 5** Multivariate analysis of association between baseline parameters and in-hospital mortality, LOHS more than seven days, and ICU treatment.

| **In-hospital death** | | | |
| --- | --- | --- | --- |
|  | **NC-CAP** | | **C-CAP** |
|  | **aOR (95% CI)** | **aOR (95% CI)** | |
| Age | **1.04 (1.01-1.08)** | **1.11 (1.05-1.18)** | |
| Female sex | 0.73 (0.28-1.70) | 0.53 (0.14-1.17) | |
| BMI | 0.95 (0.87 – 1.03) | 1.07 (0.96 – 1.18) | |
| Before pandemic | Reference | .. | |
| First wave | 2.04 (0.87-4.51) | Reference | |
| Second wave |  | **7.64 (1.58-60.60)** | |
| Third wave |  | 6.35 (0.98-58.20) | |
| Hypertension | 0.65 (0.27-1.52) | 1.53 (0.49-4.89) | |
| Atrial fibrillation | 1.18 (0.41-2.98) | 1.50 (0.25-7.19) | |
| Diabetes mellitus | 1.42 (0.48-3.70) | 1.20 (0.37-3.78) | |
| Asthma | .. | 0.73 (0.03-5.47) | |
| COPD | 0.63 (0.21-1.62) | .. | |
| Malignant disease* | **2.71 (1.19-5.99)** | **6.42 (1.44-29.91)** | |
| Dexamethasone | .. | 3.12 (0.87-12.78) | |
| Remdesivir | .. | 0.58 (0.09-2.82) | |
| Antibiotic | .. | **12.30 (3.05-73.38)** | |
| **LOHS > 7 d** | | | |
|  | **NC-CAP** | | **C-CAP** |
|  | **aOR (95% CI)** | **aOR (95% CI)** | |
| Age | **1.02 (1.01-1.03)** | **1.04 (1.01-1.06)** | |
| Female sex | 0.91 (0.69-1.19) | 0.70 (0.40-1.22) | |
| BMI | 0.98 (0.96-1.01) | 1.02 (0.97-1.08) | |
| Before pandemic | Reference | .. | |
| First wave | 0.79 (0.56-1.10) | Reference | |
| Second wave |  | **0.28 (0.13-0.58)** | |
| Third wave |  | **0.24 (0.11-0.55)** | |
| Hypertension | 1.14 (0.84-1.54) | 0.82 (0.44-1.52) | |
| Atrial fibrillation | 1.22 (0.84-1.78) | 2.08 (0.64-8.15) | |
| Diabetes mellitus | 1.27 (0.89-1.80) | 0.95 (0.47-1.96) | |
| Asthma | .. | 0.72 (0.27-1.99) | |
| COPD | 1.22 (0.90-1.66) | .. | |
| Malignant disease* | 0.97 (0.69-1.37) | 2.19 (0.70-7.80) | |
| Dexamethasone | .. | **3.43 (1.82-6.62)** | |
| Remdesivir | .. | **4.77 (2.17-11.41)** | |
| Antibiotic | .. | **3.30 (1.90-5.85)** | |
| **ICU treatment** | | | |
|  | **NC-CAP** | | **C-CAP** |
|  | **aOR (95% CI)** | **aOR (95% CI)** | |
| Age | 0.99 (0.97-1.02) | 1.01 (0.99-1.04) | |
| Female sex | 0.76 (0.38-1.43) | **0.36 (0.18-0.68)** | |
| BMI | 0.96 (0.91-1.02) | 1.03 (0.98-1.09) | |
| Before pandemic | Reference | .. | |
| First wave | 1.86 (0.94-3.52) | Reference | |
| Second wave |  | 1.13 (0.54-2.34) | |
| Third wave |  | 1.67 (0.70-3.98) | |
| Hypertension | 0.97 (0.48-1.96) | 1.19 (0.63-2.23) | |
| Atrial fibrillation | **2.23 (1.03-4.62)** | 1.47 (0.48-4.25) | |
| Diabetes mellitus | 2.11 (0.99-4.33) | 1.19 (0.60-2.33) | |
| Asthma | .. | 0.60 (0.16-1.86) | |
| COPD | 0.85 (0.38-1.72) | .. | |
| Malignant disease* | 1.30 (0.58-2.67) | 1.39 (0.42-4.28) | |
| Dexamethasone | .. | **2.17 (1.12-4.30)** | |
| Remdesivir | .. | 1.41 (0.69-2.82) | |
| Antibiotic | .. | **3.37 (1.80-6.52)** | |

Bold numbers indicate p-values < 0.05. *) Excluding patients with chemotherapy during the last three months, neutropenia, or immunosuppression after organ or stem cell transplant. ICU: intensive care unit, LOHS: length of hospital stay, COPD: chronic obstructive pulmonary disease, BMI: body mass index, C-CAP: COVID-19 community-acquired pneumonia, NC-CAP: non-COVID-19 community-acquired pneumonia, aOR: adjusted odds ratio, CI: confidence interval

**sTable 6** 180-days post-hospital admission follow-up outcomes of patients COVID-19 community-acquired pneumonia (C-CAP) and non-COVID-19 community-acquired pneumonia (NC-CAP).

|  | **NC-CAP** | **C-CAP** | **p-value** | **aOR (95% CI)** |
| --- | --- | --- | --- | --- |
| Included in follow-up (n) | 905 | 272 | .. | .. |
| Death after hospital discharge (%) | 35 (3.9) | 4 (1.5) | 0.0540 | 0.65 (0.11 - 1.99) |
| Completed follow-up (n) | 870 | 268 | .. | .. |
| Re-hospitalization during follow-up (%) | 175 (20.1) | 25 (9.3) | **0.0003** | **0.43 (0.27 – 0.70)** |

Bold numbers indicate p-values <0.05. IQR: inter-quartile range, aOR: adjusted odds ratio, CI: confidence interval.
